# Psychosocial and health behavioural characteristics of longitudinal physical activity patterns: a cohort study from adolescence to young adulthood

**DOI:** 10.1186/s12889-023-17122-4

**Published:** 2023-11-03

**Authors:** Tuula Aira, Tommi Vasankari, Olli J Heinonen, Raija Korpelainen, Jimi Kotkajuuri, Jari Parkkari, Kai Savonen, Kerttu Toivo, Arja Uusitalo, Maarit Valtonen, Jari Villberg, Henri Vähä-Ypyä, Sami P Kokko

**Affiliations:** 1 Faculty of Sport and Health Sciences, Research Centre for Health Promotion, University of Jyväskylä, PL 35, Jyväskylä, FI-40014 Finland; 2 UKK Institute of Health Promotion Research, Kaupinpuistonkatu 1, FI-33500 Tampere, Tampere, Finland; 3 Faculty of Medicine and Health Technology, Tampere University, Tampere, Finland; 4 Paavo Nurmi Centre & Unit for Health and Physical Activity, University of Turku, Kiinamyllykatu 10, FI-20520 Turku, Finland; 5 Medical Research Center (MRC), University of Oulu and University Hospital of Oulu, Oulu, Finland; 6 Department of Sports and Exercise Medicine, Oulu Deaconess Institute Foundation sr, P.O. Box 365, FI-90101 Oulu, Finland; 7 Research Unit of Population Health, University of Oulu, Oulu, Finland; 8 Department of Mathematics and Statistics, University of Jyväskylä, P.O. Box 35, FI-40014 Jyväskylä, Finland; 9 Tampere Research Center of Sports Medicine, Kaupinpuistonkatu 1, FI-33500 Tampere, Finland; 10 Faculty of Sport and Health Sciences, University of Jyväskylä, PL 35, FI-40014 Jyväskylä, Finland; 11 Department of Clinical Physiology and Nuclear Medicine, Kuopio University Hospital, Kuopio, Finland; 12 Kuopio Research Institute of Exercise Medicine, Haapaniementie 16, FI-70100 Kuopio, Finland; 13 Clinic for Sports and Exercise Medicine, Foundation for Sports and Exercise Medicine, Mäkelänkatu 47, FI-00550 Helsinki, Finland; 14 Department of Sports and Exercise Medicine, University of Helsinki, Clinicum, Finland; 15 Finnish Institute for High Performance Sport KIHU, Jyväskylä Finland, Rautpohjankatu 6, FI-40700 Jyväskylä, Finland

**Keywords:** Adolescent, Health Behaviour, Physical activity, Psychosocial factors, Young Adult

## Abstract

**Background:**

The decline in physical activity (PA) during adolescence is well-established. However, while some subgroups of adolescents follow the general pattern of decreased activity, others increase or maintain high or low activity. The correlates and determinants of different PA patterns may vary, offering valuable information for targeted health promotion. This study aimed to examine how psychosocial factors, health behaviours, and PA domains are associated with longitudinal PA patterns from adolescence to young adulthood.

**Methods:**

This prospective study encompassed 254 participants measured at mean ages 15 and 19. Device-measured moderate-to-vigorous PA was grouped into five patterns (*activity maintainers, inactivity maintainers, decreasers from moderate to low PA, decreasers from high to moderate PA, increasers*) via a data-driven method, K-Means for longitudinal data. Multinomial logistic regression was used to analyse the associations between health behaviours, psychosocial factors, PA domains, and different PA patterns.

**Results:**

A lack of sports club participation characterised *inactivity maintainers* throughout adolescence. Difficulties in communicating with one’s father at age 15 were associated with higher odds of belonging to *inactivity maintainers* and to *decreasers from moderate to low PA*. Lower fruit and vegetable consumption at age 19 was also related to increased odds of belonging to the groups of *inactivity maintainers* and *decreasers from moderate to low PA. S*moking at age 19 was associated with being a *decreaser from moderate to low PA.*

**Conclusions:**

Diverse factors characterise longitudinal PA patterns over the transition to young adulthood. Sports club participation contributes to maintained PA. Moreover, a father-adolescent relationship that supports open communication may be one determinant for sustained PA during adolescence. A healthier diet and non-smoking as a young adult are associated with more favourable PA development.

**Supplementary Information:**

The online version contains supplementary material available at 10.1186/s12889-023-17122-4.

## Background

The decline in physical activity (PA) during adolescence is a global health concern [1]. Overall, PA tends to decline with increasing age [2, 3], such that as compared to younger age groups [1], more adolescents fail to meet the one-hour moderate-to-vigorous PA (MVPA) per day recommendation [4]. However, despite the overall decline in PA, considerable individual variation exists in the development of PA over adolescent years – a point illustrated by studies identifying distinct longitudinal PA patterns or trajectories [5–9]. There is a need for studies to determine how those adolescents who maintain favourable PA differ from those who decrease their activity, or those who maintain an inactive lifestyle. Such research would open up possibilities to guide PA interventions [2, 5, 6, 10].

PA is a complex behaviour, varying over time. Numerous theories and models have been applied to shed light on it. One of these, the (socio)ecological model, is based on the idea that PA may be influenced by (1) individual (e.g. genetic or psychological) factors, (2) social or cultural factors, (3) the built environment, and (4) policies [11, 12]. Thus, the determinants and correlates of longitudinal PA patterns may exist at many levels. Moreover, the contexts of living and of PA change over the life course [13–15], and this further increases the possible correlates for sustained and changed PA. The transition to young adulthood typically involves increasing autonomy. This is frequently linked to important changes (such as moving out of the childhood family home, or entering higher education or employment) that may predispose individuals to changing their health behaviours. As well as acknowledging the changes in environments and the impacts of life events during the lifespan, the life course approach considers the impact of earlier life phases on subsequent phases, and assumes that there may be ideal times for intervening in health behaviours [16–18].

A number of possible correlates/determinants for longitudinal PA patterns during adolescence have previously been examined, including maturity [7, 19, 20], socioeconomic status [21], other health behaviours [19, 20], support for PA [20, 21], PA domains [5], and distance to the nearest park and school [20]. However, the research base is still limited, and apart from family and peer support for PA, the psychosocial factors correlating with PA patterns have received little attention. Moreover, for the most part, only a single health behaviour has been studied in relation to longitudinal PA patterns [6, 20], even if there would be reason to examine the possible accumulation of behavioural risk and/or protective factors in relation to longitudinal PA patterns. Overall, information on the co-occurring and multidimensional characteristics of longitudinal PA patterns could be valuable for health promotion, one aim of which is to increase or maintain physical activity during adolescence.

Another limitation in previous research is that only some studies have used accelerometry in PA assessment rather than self-reported measurements; furthermore, the majority of studies have not applied data-driven methods for identifying distinct PA patterns. Data-driven methods can better identify genuinely heterogeneous PA patterns as compared to subjective methods (which can involve splitting into quartiles, or applying predetermined levels of PA).

Our aim was to study how psychosocial factors (*loneliness, weight satisfaction, ease in talking to parents, exercise with parents*) and health behaviours (*alcohol, tobacco, and snuff use, amount of sleep, eating behaviours, toothbrushing*) are associated with longitudinal PA patterns from adolescence to young adulthood (referring here to the patterns represented by *activity maintainers, inactivity maintainers, decreasers from moderate to low PA, decreasers from high to moderate PA*, and *increasers* [5]). Moreover, the associations between activity domains and PA patterns were examined together with psychosocial factors and health behaviour, the aim being to assess the most important correlates for sustained and changed PA during the transition to young adulthood. In previous analyses using the same study data, changes in sports club participation have emerged as significantly related to maintained and decreased PA, and passive commuting throughout adolescence has been found to be related to maintained inactivity [5]. Thus, we hypothesised that when analysed together with the other studied factors, we would find participation in a sports club to be related to maintained or increased PA, and passive commuting to maintained inactivity. Moreover, we hypothesised that (1) decreasing PA to a low level, and (2) maintained inactivity, would be associated with at least some health-compromising behaviours and psychosocial challenges.

## Methods

### Participants and study procedure

The data for this observational cohort study were drawn from the Health Promoting Sports Club study (HPSC), conducted in the years 2013–2014 and 2017–2018. At baseline, the participants (mean age 15) were recruited on the basis of power calculations [22] from 156 sports clubs and 100 schools within six large cities and surrounding communities in different parts of Finland [5, 20].

The procedure was the same at baseline and follow-up. Hence, the participants took part in electronic surveys on current health status and behaviours, and participated in medical examinations containing screening by a physician, a fasting blood sample, and instructions to use a hip-worn accelerometer (seven consecutive days during waking hours, except when bathing or doing water activities).

Nearly two thirds (64%) of the baseline participants (*n* = 583, mean age 15.5, SD 0.6), also took part in follow-up measurements (*n* = 371, mean age 19.4, SD 0.6). In total, 254 adolescents (60% females) provided valid accelerometry data for both measurements (swimmers excluded: *n* = 22, at least four days, 10 h/day). See [5, 20] for more study details.

### Measures

#### Outcomes (longitudinal PA patterns)

PA was measured via a Hookie accelerometer (AM20 Activity Meter, Hookie Technologies Ltd., Helsinki, Finland), which collected and stored tri-axial data as actual g-units (100 Hz sampling frequency). The data were analysed in units of 6 seconds’ duration. The PA analysis was based on mean amplitude deviation analyses (MAD), calculated from a resultant tri-axial raw acceleration signal, and converted to metabolic equivalents (METs) [23, 24]. The epoch-wise MET values were further smoothed by calculating the 1-minute exponential moving average MET value for each epoch time point. MVPA was defined as ≥ 3.0 METs.

The formulation and details of the longitudinal PA patterns have been described previously [5]. In brief, by applying k-means for longitudinal data (KmL) [25] based on the two MVPA measurement periods, the participants were grouped into distinct clusters so that the clusters were as different from each other as possible. The KmL method belongs to classical algorithmic approaches (hierarchical or partitional clustering), but similar model-based methods also exist, such as mixture modelling techniques or latent class analysis [25]. In this study, the KmL was selected, as it could be run with only two measurement points. Moreover, it performs especially well when the sample size is small [26], and it is computationally less complex [27]. The longitudinal PA patterns (clusters) arrived at consisted of *inactivity maintainers, activity maintainers, decreasers from moderate (to low) PA, decreasers from high (to moderate) PA* (see Additional file 1).

#### Exposure variables

The potential correlates and determinants of interest were drawn from electronic surveys. With a few exceptions, the questions were based on the international Health Behaviour in School-aged Children (HBSC) study [1], and were repeated identically at both time points (unless otherwise stated). All the HBSC survey questions have been subject to validation and piloting at national and international levels [28], and many of the items have been assessed for their test-retest reliability [28, 29].

The questions assessing *sports club participation* and *active commuting* have been described elsewhere [5]. Dichotomous variables were used to assess participation in sports clubs and active commuting (by bike or on foot) to school (age 15), or to the study place or work (age 19). The questions assessing active commuting were modified from the *Finnish schools on the Move* survey [30].

*Psychosocial variables*: *Loneliness* was measured by one question: ‘Do you ever feel lonely?’ In further categorisation, the response options *very often* and *often* were combined, as were *sometimes* and *never*. A question estimating *satisfaction with one’s own current weight* included two response options (*yes/no*), and it was created for the purposes of the present study.

*Communication with parents*: The study participants were asked how easy it was for them to talk to their mother about things that really bothered them. The response options were: *very easy, easy, difficult*, and *very difficult*, with the additional response option *I don’t have or see this person*. Identical questions were posed regarding communication with the father and (if applicable) stepfather and stepmother. Separate dichotomised variables (*easy* vs. *difficult*) were formed for communication with (1) the mother and (2) the father. The latter also included talking over difficulties with one’s stepfather in cases where the respondent did not have a father (baseline *n* = 3, follow-up *n* = 5). Cases were excluded where there was neither mother nor stepmother (baseline *n* = 3, follow-up *n* = 8) (and similarly neither father nor stepfather, baseline *n* = 14, follow-up *n* = 16).

The frequency of *exercising together with a parent* was assessed via a question: ‘During a typical week: How often does your mother (or your stepmother if your mother does not live in your primary home)…. exercise or do sport with you?’ A corresponding question assessed exercising together with the father. Two categories were formed, encompassing (1) *sometimes* to *very often*, and (2) *never* to *occasionally*. Category 2 also included not having the parent in question, or not seeing that parent.

*Health behaviour*: The *frequency of alcohol consumption* was based on a question used in the Finnish School Health Promotion (SHP) study [31]: ‘On the whole, how often do you consume alcohol, for example a half-bottle of beer or more?’. The response options ranged from *Once a week or more often* to *I don’t drink alcoholic beverages*. A dichotomised variable was formed: (1) *at least once a month*, (2) *less frequently or no consumption at all*.

*Lifetime drunkenness* was based on a question asking adolescents whether they had ever had so much alcohol that they were really drunk. The response alternatives ranged from *never* to *more than 10 times*. A dichotomised variable was formed for lifetime drunkenness: (1) *two times or more* vs. (2) *never or once*.

*Snuff use* was assessed via the following question ‘Do you currently use snuff?’ The responses were categorised into two groups encompassing (1) *less frequently* to *every day*, (2) *non-users*. A correspondingly dichotomised variable was used to assess *smoking frequency*. Here, the categories *less than weekly, weekly*, and *daily smokers* were combined, due to the overall small proportion of current daily smokers at age 15.

*Toothbrushing frequency* was determined via a question: ‘How often do you brush your teeth?’. The answer options were dichotomised (*< twice daily* vs. *twice daily*) according to the international recommendation of twice-daily toothbrushing. Furthermore, the responses on *frequency of eating breakfast on weekdays* and *frequency of eating school meals* were dichotomised to (*5 days* vs. *4 days or less per week*).

The *fruit and vegetable index* and the *sweets and sugared soft drinks index* were based on a question assessing the consumption of listed foods and drinks. The response options ranged from *never* to *every day, more than once*. The vegetable index ranged from *0* to *14*, where value 0 represented *no fruit and vegetable consumption*, and value 14 *consumption of both fruit and vegetables at least once a day.* Correspondingly, value 0 indicated *consuming both sweets and sugared soft drinks at least once a day*, while value 14 indicated *never eating sweets and sugared soft drinks* (for more details see 32, 33). Energy drinks (with examples given such as Battery, RedBull) were also among the listed foods and drinks, and adolescents’ responses indicating *energy drink consumption* were categorised into two groups: (1) *at least weekly*, (2) *never or less than weekly*.

*The amount of sleep* was asked by a question created for the purposes of the present study: ‘How many hours do you sleep on average on weekdays?’. Respondents reported the amount by a number.

### Data analysis

The cross-sectional differences between PA patterns were assessed with cross-tabulations and Chi-square test/Fisher’s exact test for categorial variables, and with the Kruskall-Wallis test (with post hoc Dunn’s test, adjusted by the Bonferroni correction for multiple tests) for continuous variables.

Multinomial logistic regression analyses were conducted to calculate odds ratios (ORs) with 95% confidence intervals (CIs) for the associations between the exposure variables and membership of PA patterns. For these analyses, the categories of *activity maintainers* and *increasers* were combined due to the relatively small number of increasers (*n* = 20), and because both categories represented a favourable evolution of PA in terms of health. This combined group was used as a reference in the analyses. Multiple different models were tested with the forced entry method, until the best-fitting ones were reached separately for (1) the baseline (mean age 15) and (2) the follow-up (mean age 19). Thus, the models (1) predicted membership of each pattern by determinants from the baseline (mean age 15), and (2) characterised the patterns at mean age 19 (longitudinal correlates). The models were adjusted for the *measurement interval* (age at the 2nd measurement minus age at the 1st measurement) and for the *change in device wear-time*. Missing cases (*n* = 1–14) were excluded from the analyses. The data analysis was performed using SPSS version 26, and the significance level was set at *p* < 0.05 in all the statistical tests.

## Results

### Descriptive information

Detailed descriptive information on the study participants has been provided previously [5], see also Additional file 2. Briefly, at baseline, nearly two-thirds (62%) of the participants lived in families with high affluence. At follow-up, most of the participants (69%) were still living with their parents. Half of the sample (49%) were studying in upper secondary education, while 13% were studying in higher education, and 21% were working (not studying) during the follow-up measurement. The *inactivity maintainers* were mostly females (73%), while the *decreasers from high to moderate* were mostly males (vs. 19% females). There were no other differences in sociodemographic characteristics between the PA patterns (see Additional file 2).

A previous study found no differences in baseline MVPA, family affluence, or perceived health between the baseline participants and those lost to follow-up [5]. However, males, and those who reported lower school achievement, were more likely not to participate in the post-measurement (*p* < 0.001).

### Psychosocial and health behavioural characteristics of the PA patterns: univariable analysis

As shown in Tables 1 and 2, there were differences between PA patterns in multiple psychosocial variables (loneliness, communication difficulties with father, exercise with parents, and weight satisfaction) and in some health behaviours (smoking, dietary habits, and sleep). For example, *exercising together with a parent at least occasionally* was less frequent among *inactivity maintainers* (57%) and *decreasers from moderate to low* (59%) at age 15 as compared to *increasers* (95%) (Table 1). Health behaviours did not differ between PA patterns at age 15, apart from a slightly lower average sleep amount among *inactivity maintainers* as compared to *decreasers from high to moderate PA* and *increasers* (Table 2).

**Table 1:** Psychosocial characteristics compared between longitudinal physical activity patterns, *n* (%)

	All	Inactivity maintainers	Activity maintainers	Decreasers from moderate PA	Decreasers from high PA	Increasers	*p*-value
Feeling loneliness quite often/often							
age 15	15 (6)	6 (9)	4 (6)	2 (3)	2 (7)	1 (5)	0.778
age 19	24 (10)	10 (14)	4 (6)	10 (16)	0	0	0.017
Ease of talking with mother							
age 15	202 (82)	57 (84)	57 (84)	44 (73)	26 (87)	18 (90)	0.390
age 19	208 (83)	57 (81)	54 (77)	49 (82)	30 (94)	18 (95)	0.182
Ease of talking with father							
age 15	163 (68)	38 (57)	49 (77)	33 (55)	26 (90)	17 (85)	< 0.001
age 19	153 (64)	41 (61)	37 (56)	34 (60)	24 (83)	17 (90)	0.012
Ease of talking with at least one parent							
age 15	209 (85)	58 (85)	61 (88)	46 (77)	26 (87)	18 (90)	0.434
age 19	217 (86)	60 (86)	56 (80)	52 (85)	30 (94)	19 (100)	0.139
Mother exercises or does sport with you at least occasionally							
age 15	106 (43)	27 (40)	35 (51)	20 (33)	10 (33)	14 (70)	0.020
age 19	88 (35)	18 (26)	30 (43)	22 (36)	5 (16)	13 (68)	0.001
Father exercises or does sport with you at least occasionally							
age 15	120 (48)	21 (31)	38 (55)	25 (41)	17 (57)	19 (95)	< 0.001
age 19	73 (29)	16 (23)	24 (35)	15 (25)	7 (23)	11 (58)	0.024
At least one parent exercises or does sport with you							
age 15	162 (65)	39 (57)	49 (71)	36 (59)	19 (63)	19 (95)	0.010
age 19	119 (47)	30 (43)	40 (57)	29 (48)	7 (23)	13 (68)	0.006
Satisfied with one’s own weight							
age 15	194 (77)	46 (67)	51 (74)	47 (77)	31 (97)	19 (95)	0.002
age 19	179 (72)	41 (59)	51 (73)	40 (69)	30 (94)	17 (85)	0.003

Note: The identification of the longitudinal physical activity patterns was based on two valid accelerometry measurement periods of moderate-to-vigorous physical activity, and used a data-driven method (K-means for longitudinal data, KmL [25]) [5]. *p*-values have been assessed using the Chi-square test or Fisher’s exact test (in cases of sparse data) for categorical variables

**Table 2:** Health behavioural characteristics compared between longitudinal physical activity patterns

	Mean age	All	Inactivity maintainers (A)	Activity maintainers (B)	Decreasers from moderate PA (C)	Decreasers from high PA (D)	Increasers (E)	*p*-value
Smoking (other than non-smoking), *n* (%)	15	14 (6)	6 (9)	3 (4)	5(8)	0	0	0.333
	19	34 (13)	14 (20)	6 (9)	12 (20)	2 (6)	0	0.036
Snuff use (other than no snuff use), *n* (%)	15	8 (3)	4 (6)	1 (1)	2 (3)	0	1 (5)	0.469
	19	25 (10)	6 (9)	4 (6)	7 (12)	7 (22)	1 (5)	0.155
Alcohol consumption at least once a month, *n* (%)	15	21 (9)	9 (13)	6 (9)	4 (7)	2 (7)	0	0.453
	19	167 (66)	45 (63)	49 (70)	36 (59)	24 (75)	13 (68)	0.533
Have been drunk at least twice, *n* (%)	15	25 (10)	11 (16)	8 (12)	3 (5)	2 (7)	1 (5)	0.268
	19	166 (66)	47 (66)	42 (60)	39 (64)	26 (81)	12 (63)	0.333
Eating a school meal every school day, *n* (%)	15	204 (82)	58 (85)	56 (81)	51 (84)	22 (73)	17 (85)	0.694
Eating breakfast every weekday morning, *n* (%)	15	165 (67)	42 (62)	48 (70)	40 (66)	21 (70)	14 (70)	0.866
	19	209 (83)	53 (76)	57 (81)	51 (84)	30 (94)	18 (95)	0.134
Daily consumption of fruits or/and vegetables, *n* (%)	15	110 (44)	26 (38)	36 (52)	23 (38)	15 (50)	10 (50)	0.348
	19	108 (43)	23 (32)	37 (53)	21 (34)	18 (56)	9 (47)	0.035
Fruit and vegetable consumption index, mean (SD)	15	9.1 (4.3)	8.0 (4.6)	9.9 (3.9)	8.5 (4.4)	10.6 (3.7)	10.0 (4.2)	ns
	19	9.3 (4.0)	7.7 (4.2)	10.6 (3.6)	8.7 (4.1)	10.8 (3.0)	10.0 (4.0)	A < B** A < D**
Sweets and sugared soft drinks consumption index, mean (SD)	15	6.0 (3.5)	5.9 (3.6)	6.1 (3.5)	6.6 (3.2)	4.9 (3.4)	5.9 (3.5)	0.240
	19	7.2 (3.4)	7.1 (3.4)	7.2 (3.6)	7.3 (3.0)	7.7 (3.7)	6.7 (3.5)	0.915
At least weekly energy drink consumption, *n* (%)	15	20 (8)	8 (12)	4 (6)	4 (7)	2 (7)	2 (10)	0.721
	19	27 (11)	5 (7)	7 (10)	4 (7)	8 (25)	3 (16)	0.065
Brushing teeth two times per day, *n* (%)	15	158 (64)	44 (65)	45 (65)	40 (66)	19 (63)	10 (50)	0.769
	19	174 (69)	48 (68)	49 (70)	40 (66)	24 (75)	13 (68)	0.915
Sleep duration on weekdays, mean hours (SD)	15	8.2 (0.8)	8.0 (0.8)	8.3 (0.7)	8.3 (0.8)	8.5 (0.6)	8.6 (0.8)	A < D* A < E*
	19	7.8 (0.9)	7.7 (0.9)	7.7 (1.0)	7.8 (0.9)	7.8 (0.8)	7.9 (0.6)	ns

Note: The identification of the longitudinal physical activity patterns was based on two valid accelerometry measurement periods of moderate-to-vigorous physical activity, and used a data-driven method (K-means for longitudinal data, KmL [25]) [5]. *p*-values have been assessed using the Chi-square test or Fisher’s exact test (in cases of sparse data) for categorical variables. The Kruskal-Wallis test was used in analysing differences in mean values between PA patterns cross-sectionally (with post hoc Dunn’s test adjusted by the Bonferroni correction for multiple tests). ns = non significant; PA = physical activity; SD = standard deviation; * *p* < 0.05; ** *p* < 0.01

### Correlates and determinants (multivariable analysis)

**Fig. 1:**
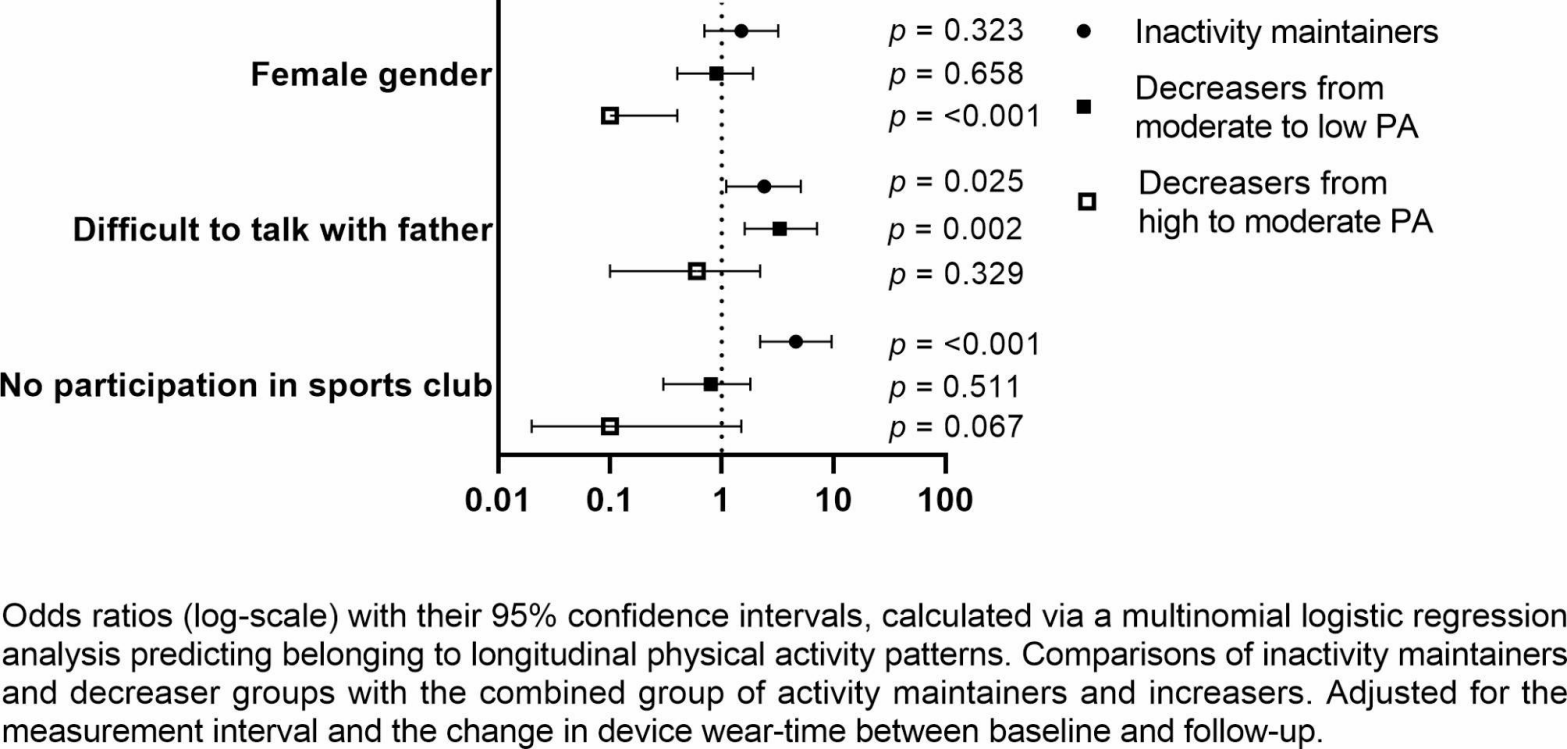
Analysis stemming from exposure variables presented at baseline (mean age 15)

**Fig. 2:**
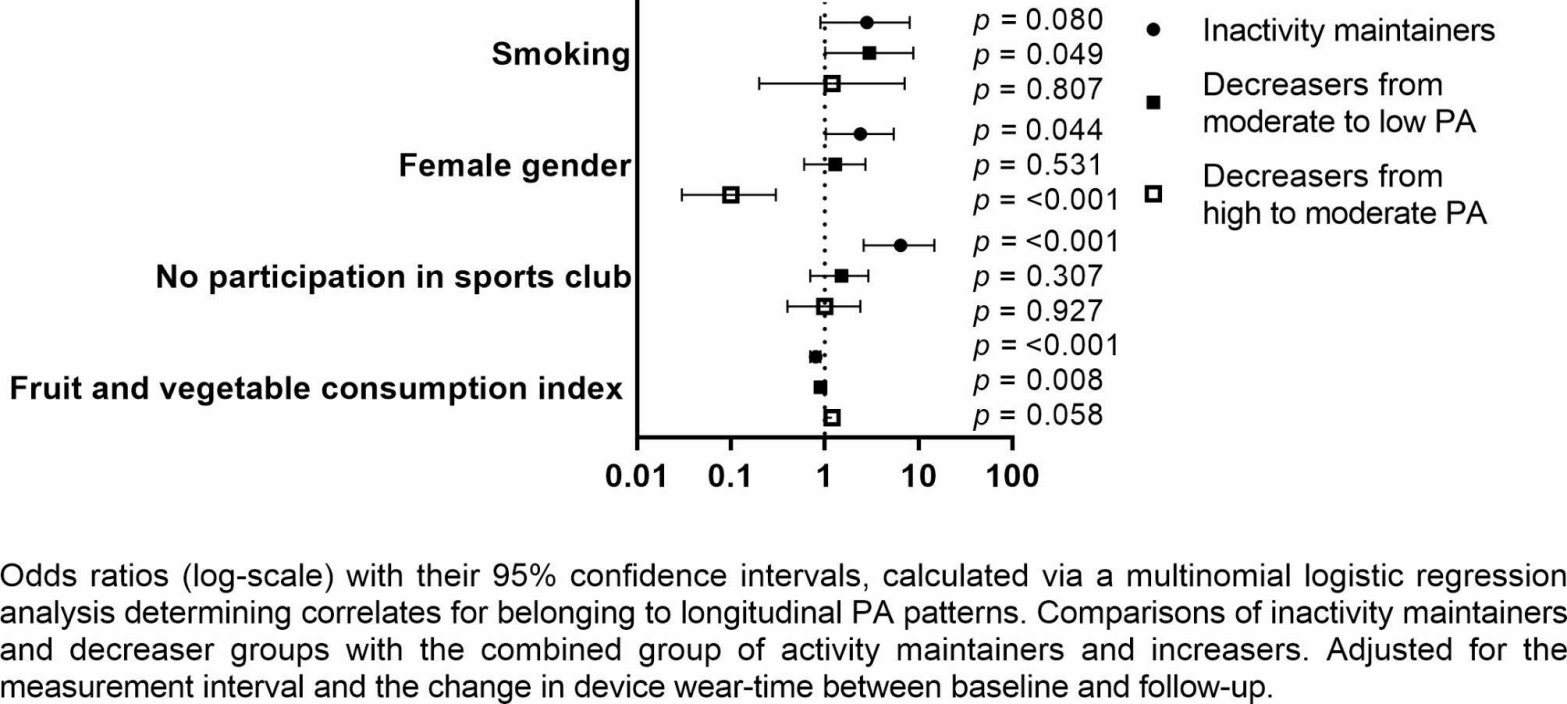
Analysis stemming from exposure variables presented at follow-up (mean age 19)

The most significant correlates and determinants for belonging to PA patterns were detected via multinomial logistic regression, using the combined group of *activity maintainers* and *increasers* as a reference (Figs. 1 and 2, Additional file 3). Both of the best-fitting models included *sports club participation* and *gender* as the most significant exposure variables, together with *talking about difficulties with one’s father* (using a model consisting of only baseline predictors) and *fruit and vegetable consumption* (using a model consisting of only exposure variables from follow-up measurements).

*Not participating in a sports club* (OR: 4.6; CI: 2.2–9.6) and *communication difficulties with one’s father* (OR: 2.4; CI: 1.1–5.1) at age 15 were associated with increased odds of being an *inactivity maintainer* as compared to membership of a group with favourable PA development. Correspondingly, *communication difficulties with one’s father* at age 15 (OR: 3.3; CI: 1.6–7.1) was related to belonging to *decreasers from moderate to low PA*. *Male gender* was the only significant determinant for belonging to the *decreasers from high PA*.

*Female gender* (OR: 2.4; CI: 1.03–5.4), *lower fruit and vegetable consumption* (OR: 0.8; CI: 0.7–0.9), and *not participating in a sports club at age 19* (OR: 6.4; CI: 2.6–14.7) were associated with increased odds of being an *inactivity maintainer* as compared to belonging to the combined group of *activity maintainers* and *increasers*. A *lower fruit and vegetable consumption index at age 19* (OR: 0.9; CI: 0.8–0.97) was also related to being a *decreaser from moderate PA*. The same was true of *smoking* (OR: 3.0; 1.005–8.8).

## Discussion

The study aimed to determine the psychosocial, health behavioural and PA domain-related characteristics of different longitudinal PA patterns from adolescence to young adulthood. The patterns (i.e. groups with different PA development over time) were based on device-measured MVPA and formed via a novel data-driven method. Multilevel factors were found to characterise the PA patterns. At age 15, *gender, sports club participation*, and *communication difficulties with one’s father* were the most important predictors for PA patterns. Correspondingly, at age 19, *gender, sports club participation*, and *fruit and vegetable consumption* were associated with longitudinal PA patterns.

A lack of sports club participation characterised *inactivity maintainers* throughout adolescence. Indeed, such non-participation showed the strongest association for belonging to this PA pattern. The result is in line with previous studies showing the relation between sports participation and maintained PA during adolescence [5, 9, 19]. Sports clubs have an important role in supporting maintained PA, including during early young adulthood, even if dropout from organised sports is common during adolescence [5, 34]. In Finland, sports clubs are typically based on voluntary civic activities at local level.

Surprisingly, *communication difficulties with the father* (but not with the mother) were related to increased odds of being a *decreaser from moderate to low PA* and also of being an *inactivity maintainer.* This implies that a father-adolescent relationship that supports open communication might be one determinant for sustained PA behaviour during adolescence. Note that we used only one question to assess the broad phenomenon, and that the association disappeared at age 19. One can suggest that analysis of the communication and relationship with parents would be a topic for future research on PA development during adolescence, since previous research has shown that parent-adolescent communication predicts adolescent MVPA one year later [35], and that communication difficulties pose a risk for decreased life-satisfaction [36–38]. Generally speaking, there has been a positive increasing trend in ease of communication with both parents over the 2000s, although communication with the mother has appeared to be easier than with the father [39, 40]. Future studies should examine whether communication with friends (including girl-/boyfriend) and siblings compensates for poorer communication with parents, especially during young adulthood. Further research could also encompass the extent to which a parent’s divorce (and subsequent residential arrangements) and the adolescent’s individual temperament alter the association between communication problems and PA patterns.

Feelings of *loneliness* at age 19 were more commonly reported by *inactivity maintainers* (14%) and by *decreasers from moderate to low PA* (16%) as compared to *increasers* (0%) and *decreasers from high to moderate PA* (0%). Because frequent loneliness was non-existent in some PA patterns, the loneliness variable did not fit into the logistic regression models. The association we found in cross tabulation is logical also when placed alongside a review study which provided evidence for the potential diminishing effect of loneliness on PA – but conversely, the potential value of PA in reducing loneliness [41].

From the multiple health behaviours analysed only *fruit and vegetable consumption* and *smoking* appeared among the multilevel correlates for PA patterns at age 19. Lower fruit and vegetable consumption was related to increased odds of belonging to both *inactivity maintainers* and *decreasers from moderate to low PA*, with smoking also being related to the decreaser group. These findings are more or less as expected, since both unhealthy and harmful behaviours tend to accumulate [42]. Moreover, the results here are in line with previous research indicating that fruit and vegetable consumption is higher among adolescents who are persistently active [43]. In another study exploring multiple health behaviours (but not dietary habits), smoking, and being drunk (the latter only for girls) at baseline (i.e. among 13- to 15-year-olds) were associated with increased odds of being an inactivity maintainer rather than an activity maintainer [19]. Similarly, smoking at a young adult age was more common among persistently sedentary participants than among their persistently active counterparts [44]. In our study, neither alcohol consumption nor binge drinking differed between the PA patterns, but the results are similar to previous studies insofar as smoking seems to be related to unfavourable PA development over time. According to a recent review study – which did not consider different longitudinal PA patterns – PA is positively associated with alcohol use among emerging adults (aged 18–25) but less consistently among adolescents [45]. However, methodological and contextual differences hinder comparison between the studies in question.

*The decreasers from high to moderate PA* constituted a relatively small group that could be characterised only by male gender. Maintained participation in sports clubs and also withdrawal from sports clubs (as opposed to ‘never-participation’, i.e. non-participation at either age 15 or 19) have previously been related to increased odds of being in this group of decreasers [5]. However, as compared to the other PA patterns detected, this decreaser group is less homogeneous, as shown by the relatively wide standard deviation in PA levels. These limit the possibility to form conclusions on the characteristics of this pattern. A similar pattern, involving a substantial decrease from high MVPA, has been found in a previous study by Kwon et al. [21], in which the male gender was more prevalent.

With regard to the other studied background variables, a lack of active commuting at either baseline or follow-up did not significantly differentiate between the PA patterns, even if sustained passive commuting has been related to maintained inactivity in the same study population (when inactive maintainers were compared to all the other groups together) [5]. This finding is a reminder that the time-varying nature of possible correlates should be also taken into account, and not just the associations at a single time point (see also [18, 46]).

### Strengths and limitations

The main strengths of this study are the use of relatively large prospective data from a less-studied period of life (see also [2, 3, 6]), combining information from both survey and accelerometry to assess determinants and correlates for changed and maintained PA. Instead of grouping participants on the basis of predetermined PA levels, we used a novel data-driven method to identify genuinely heterogeneous PA patterns. The survey questions were mainly drawn from the international HBSC study, and the questions have thus been subject to validation [28].

The study encompassed multilevel predictors and correlates. However, it remains possible that certain other factors, operating for instance at the environmental level, might determine the differences between PA patterns. The data were limited to two measurements, and additional time points might have revealed more fluctuations in PA during adolescence. Furthermore, the number of persons in some of the PA patterns was small, which decreased the statistical power. Partly because of the sampling method (involving both sports clubs and schools), the young people in this study were more active than on average. However, this is also a strength, since it enabled a thorough analysis of PA and related factors for highly active young people in addition to those in the average population. Moreover, it can be claimed that the recruitment of the participants from six different regions of Finland (from 100 schools and 156 sports clubs) increased the quality of this study, even if the results are not directly generalisable to the entire Finnish age cohort in question.

## Conclusions

This study contributed to the limited research base on PA development and its correlates during the transition to young adulthood. The results indicate that the relationship with one’s father may play a role in supporting maintained PA during the adolescent years. Furthermore, a healthier diet and non-smoking as a young adult are associated with more favourable PA development. The study gives indications that although dropout from a sports club is common during adolescence [5, 34], organised sports still have a contribution in supporting favourable PA development when adolescents reach young adulthood.

While the study was successful in revealing some characteristics of the PA patterns, much variation exists in PA behaviour and related factors. Thus, the findings also demonstrate the complex and multidimensional nature of PA behaviour. This implies that there can be no ‘one size fits all’ in PA promotion; nevertheless, by addressing multiple different aspects, there may be better possibilities for preventing a decrease in PA and supporting the maintenance of healthy PA.

### Electronic supplementary material

Below is the link to the electronic supplementary material.

Supplementary Material 1

Supplementary Material 2

Supplementary Material 3

## Data Availability

The data underlying this article cannot be shared publicly since they contain confidential personal details and health information. The data will be shared on reasonable request to the principal investigator (sami.p.kokko@jyu.fi).

## Abbreviations

CI: confidence interval
MET: metabolic equivalent
MVPA: moderate-to-vigorous physical activity
OR: odds ratio
PA: physical activity
