# Supplementary material for: Psychosocial and health behavioural characteristics of longitudinal physical activity patterns: a cohort study from adolescence to young adulthood
Source: BMC Public Health. 2023 Nov 3;23:2156. doi: 10.1186/s12889-023-17122-4 (PMC10625285; doi:10.1186/s12889-023-17122-4)
Supplement: Supplementary file 1 — Supplementary Material 1 [file 12889_2023_17122_MOESM1_ESM.docx]

**Additional file 1**





**Supplementary Figure 1**. Longitudinal physical activity patterns (the data show the mean and the standard deviation)

**References:**

Aira T, Vasankari T, Heinonen OJ, et al. Physical activity from adolescence to young adulthood: patterns of change, and their associations with activity domains and sedentary time. Int J Behav Nutr Phys Act. 2021; doi:10.1186/s12966-021-01130-x

Aira T, Kokko S, Heinonen OJ, et al. Longitudinal physical activity patterns and the development of cardiometabolic risk factors during adolescence. Scan J Med Sci Sports. 2023. doi: 10.1111/sms.14415
