# Supplementary material for: Psychosocial and health behavioural characteristics of longitudinal physical activity patterns: a cohort study from adolescence to young adulthood
Source: BMC Public Health. 2023 Nov 3;23:2156. doi: 10.1186/s12889-023-17122-4 (PMC10625285; doi:10.1186/s12889-023-17122-4)
Supplement: Supplementary file 2 — Supplementary Material 2 [file 12889_2023_17122_MOESM2_ESM.docx]

**Additional file 2.**

**Supplementary Table 1.** Descriptive information of the physical activity patterns

|  | All | Inactivity maintainers | Activity maintainers | Decreasers from  moderate PA | Decreasers from high PA | Increasers | *p* |
| --- | --- | --- | --- | --- | --- | --- | --- |
| Gender, females, *n* (%) | 153 (60) | 52 (73) | 45 (64) | 39 (64) | 6 (19) | 11 (55) | <0.001 |
| Age, mean years (SD) |  |  |  |  |  |  |  |
| 1^st^ measurememt | 15.5 (0.6) | 15.6 (0.5) | 15.5 (0.6) | 15.3 (0.5) | 15.5 (0.5) | 15.4 (0.5) | 0.138 |
| 2^nd^ measurement | 19.4 (0.6) | 19.4 (0.7) | 19.4 (0.7) | 19.2 (0.7) | 19.5 (0.6) | 19.5 (0.5) | 0.130 |
| High family affluence^a^ (age 15), *n* (%) | 154 (62) | 38 (56) | 44 (64) | 38 (62) | 22 (73) | 12 (60) | 0.589 |
| Self-reported school grade average: good to excellent (age 15) (grades 8–10 in grading 4–10), *n* (%) | 205 (83) | 52 (77) | 57 (83) | 52 (85) | 27 (90) | 17 (85) | 0.556 |
| Living in urban area, *n* (%) |  |  |  |  |  |  |  |
| age 15 | 161 (65) | 35 (52) | 49 (71) | 42 (69) | 21 (70) | 14 (70) | 0.111 |
| age 19 | 198 (78) | 51 (72) | 58 (83) | 49 (80) | 27 (84) | 13 (68) | 0.339 |
| Education and employment status (age 19), *n* (%) |  |  |  |  |  |  |  |
| Studying | 160 (63) | 40 (56) | 47 (67) | 43 (71) | 19 (59) | 10 (53) | 0.503^b^ |
| in general upper secondary school | 100 (40) |  |  |  |  |  |  |
| in vocational school | 22 (9) |  |  |  |  |  |  |
| in higher education institution^c^ | 34 (13) |  |  |  |  |  |  |
| Working | 54 (21) | 18 (25) | 13 (19) | 9 (15) | 8 (25) | 5 (26) |  |
| Other | 39 (15) | 13 (18) | 10 (14) | 9 (15) | 5 (16) | 4 (21) |  |
| unemployed/temporarily laid off | 17 (6) |  |  |  |  |  |  |
| doing military service | 10 (4) |  |  |  |  |  |  |
| Living with parents (age 19), *n* (%) | 174 (69) | 50 (70) | 48 (69) | 39 (64) | 23 (72) | 14 (74) | 0.892 |

Note: Statistically significant *p*-values are in bold; *p*-values have been assessed using Chi-square test or Fisher exact test (in cases of sparse data) for categorical variables. The Kruskal-Wallis test was used in analysing differences in mean values between PA patterns cross-sectionally (post hoc Dunn’s test adjusted by the Bonferroni correction for multiple tests). The information of this table has been published previously, see [1].

^a^ The assessment of family affluence is based on adolescents’ answers to questions on four common consumption indicators of material deprivation (cars, bedrooms, computers, vacations) [2]. A composite Family Affluence Scale score was calculated for each youth based on his or her responses to these four items. The scale has previously been validated in the Health Behaviour in School-aged Children study [3].

^b^ The *p-*value represents the difference between the groups in bold font (studying, working, other) and PA patterns.

^c^ University or university of applied sciences

**References**

1. Aira T, Vasankari T, Heinonen OJ, et al. Physical activity from adolescence to young adulthood: patterns of change, and their associations with activity domains and sedentary time. Int J Behav Nutr Phys Act. 2021; doi:10.1186/s12966-021-01130-x

2. Currie C, Nic Gabhainn S, Godeau E, Roberts C, Smith R, Currie D, et al. Editors. Inequalities in young people’s health. HBSC international report from the 2005/2006 survey. Health policy for children and adolescents, no 5. Copenhagen: WHO regional office for Europe; 2008.

3. Boyce W, Torsheim T, Currie C, Zambon A. The family affluence scale as a measure of national wealth: validation of an adolescent self-report measure. Soc Indic Res. 2006; doi:10.1007/s11205-005-1607-6.
