# Supplementary material for: Psychosocial and health behavioural characteristics of longitudinal physical activity patterns: a cohort study from adolescence to young adulthood
Source: BMC Public Health. 2023 Nov 3;23:2156. doi: 10.1186/s12889-023-17122-4 (PMC10625285; doi:10.1186/s12889-023-17122-4)
Supplement: Supplementary file 3 — Supplementary Material 3 [file 12889_2023_17122_MOESM3_ESM.docx]

**Supplementary Table 2.** Baseline and follow-up determinants/correlates relating to longitudinal PA patterns; with odds ratios (95% confidence intervals), *p-*values, and results from model statistics (via multinomial logistic regression analysis)

| Baseline (mean age 15) exposure variables | Inactivity maintainers | | Decreasers from moderate PA | | Decreasers from high PA | |
| --- | --- | --- | --- | --- | --- | --- |
|  | OR (95% CI) | *p* | OR (95% CI) | *p* | OR (95% CI) | *p* |
| No participation in sports club | 4.6 (2.2–9.6) | <0.001 | 0.8 (0.3–1.8) | 0.511 | 0.1 (0.02–1.5) | 0.067 |
| Difficult to talk to father | 2.4 (1.1–5.1) | 0.025 | 3.3 (1.6–7.1) | 0.002 | 0.6 (0.1–2.2) | 0.392 |
| Female gender | 1.5 (0.7–3.2) | 0.323 | 0.9 (0.4–1.8) | 0.658 | 0.1 (0.1–0.4) | <0.001 |
| Model statistics: | | | | | | |
| R² Nagelkerke | 0.362 | | | | | |
| R² Cox&Snell | 0.337 | | | | | |
|  |  | | | | | |
| Follow-up (mean age 19) exposure variables | Inactivity maintainers | | Decreasers from moderate PA | | Decreasers from high PA | |
|  | OR (95% CI) | *p* | OR (95% CI) | *p* | OR (95% CI) | *p* |
| Fruit and vegetable consumption index | 0.8 (0.7–0.9) | <0.001 | 0.9 (0.8–0.97) | 0.008 | 1.2 (1.0–1.3) | 0.058 |
| No participation in sports club | 6.4 (2.6–14.7) | <0.001 | 1.5 (0.7–2.9) | 0.307 | 1.0 (0.4–2.4) | 0.927 |
| Female gender | 2.4 (1.03–5.4) | 0.044 | 1.3 (0.6–2.7) | 0.531 | 0.1 (0.03–0.3) | <0.001 |
| Smoking | 2.8 (0.9-8.0) | 0.080 | 3.0 (1.005-8.8) | 0.049 | 1.2 (0.2-7.1) | 0.807 |
| Model statistics: |  | | | | | |
| R² Nagelkerke | 0.382 | | | | | |
| R² Cox&Snell | 0.356 | | | | | |

Note: The combined group of *activity maintainers* and *increasers* was the reference. The models were adjusted for the measurement interval and the change in the device wear-time between baseline and follow-up.
